# Physical–Chemical Composition and Quality Related Changes in “Ruaner” Pear (*Pyrus ussuriensis*) During Freezing–Thawing Period

**DOI:** 10.3390/molecules24142611

**Published:** 2019-07-18

**Authors:** Yulian Liu, Yuxia Wu, Fei Che, Zhimin Zhang, Baihong Chen

**Affiliations:** 1 College of Horticulture, Gansu Agricultural University, Lanzhou 730070, China; 2 Sichuan Kaijiang Middle School, Dazhou 63625, China; 3 College of Agroforestry Engineering and Planning, Tongren University, Tongren 554300, China

**Keywords:** freezing–thawing, quality, physical–chemical composition, “Ruaner” pear

## Abstract

“Ruaner” pear (*Pyrus ussuriensis* Maxim.) is a fruit crop that is frequently served frozen in China. It is a typical postharvest ripening fruit that needs to ripen after harvest before it can be eaten, and freezing–thawing is one way that pears are treated during postharvest ripening. In order to study the physical–chemical composition and quality-related changes in “Ruaner” pears that result the freezing–thawing period, “Ruaner” pears were kept in a freezer (−20 °C) for 7 days, after which they were transferred to room temperature for thawing. The color of the peel of the “Ruaner” pears changed from yellow-green to yellow and then brown. The chlorophyll content and titratable acidity (TA) decreased significantly throughout 0–12 h period. The carotenoid content tended to rise and then decrease, peaking at 3 h after thawing (HAT), while the soluble solids content (SSC), firmness, total phenolic content, and total flavonoid content all generally decreased. The composition of soluble sugars and organic acids was examined in “Ruaner” pears, and the major soluble sugars were fructose and glucose, with citric acid being the most abundant organic acid. The data suggest that freezing–thawing significantly changes firmness, water content, SSC, and TA in “Ruaner” pears. At 3–4 HAT, “Ruaner” pears have moderate hardness, high water content, low acid content, and higher total phenolic, total flavonoid, and soluble solids content. Therefore, 3–4 HAT is the best time for pears in terms of both table and processing quality.

## 1. Introduction

Pear is one of the most important fruit crops, and there are five groups of commercial pear cultivars in the world [1]. In China, the Ussurian pear species is distributed in northern China, the northern part of Hebei, along the Yellow River and Hexi Corridor, and so on. Some wild populations of Ussurian typically grow in the cold and dry river valleys of the mountains. It can endure temperatures as cold as −45 to −52 °C [2]. Cold storage is the most common storage method for pears. In the north of China, a traditional and natural storage method is used to produce frozen fruit, and frozen pear is one of the most popular frozen fruits [3]. Ussurian pears are round or oblate, yellowish green, and calyx persistent; they have coarse flesh and abundant sclereid in the sarcocarp [4]. During the freezing–thawing stage, the pears become yellow and then brown while becoming more delicious and more fragrant. Compared with fresh pears, the total acid content decreases by about 12.5%, while the soluble solids and glucose of frozen pears significantly increase by about 20% [3].

Fruit ripening is a complex physiological and biochemical process that involves changes in color, firmness, sugar acid components, aroma, and so on, and these indexes are also important factors affecting fruit flavor, especially soluble sugars, organic acids, and sugars/acids. Softening is a characteristic of the ripening process common to most fruits. It is shown to be associated with the activity wall-degrading enzymes and chemical composition of cell [5]. In the early stage of fruit development, the accumulation of carbohydrate is mainly starch, which is converted into soluble sugars in the later stage [6,7], resulting in a decrease in cell swelling and softening of the fruit [8,9,10]. Thus, soluble sugars and starch metabolism play important roles in the process of ripening. However, sugar accumulation patterns and concentrations differ among species. In most fruits, glucose, fructose, sucrose, and sorbitol form the major proportion of soluble sugars, whereas fructose is the predominant sugar in pears and apples [11,12,13]. The major organic acids in pears include malic acid and citric acid [14,15,16]. These are important to the pear fruit’s taste, which depends on the content and type of soluble sugars and organic acids [17,18].

“Ruaner” pear is a typical postharvest ripening fruit. In China, differently to the consumption forms of most pears, consumers in the “Ruaner” pear producing areas freeze the pears during the postharvest ripening process, which is convenient for storage and improves the edible quality and taste of the “Ruaner” pear fruits. Some studies have examined the postharvest ripening process of “Ruaner” pear [19,20], but most of them have focused on room-temperature postharvest ripening. Thus, we need a better understanding of the physical–chemical composition and quality-related changes in “Ruaner” pear fruit during the freezing–thawing process. Understanding these aspects of “Ruaner” pear could potentially improve our understanding of the fundamental biological processes in agriculturally important fruit plants. Therefore, we investigated the development and changes in the pigments, firmness, water content, and composition of polyphenols, flavonoids, soluble sugars, and organic acids in “Ruaner” pear fruit to understand the development of the fruit’s quality and to provide information about the composition and concentration of metabolites to processors and consumers of this cultivar.

## 2. Results

### 2.1. Variation in Pigments in “Ruaner” Pear

**Figure 1:**
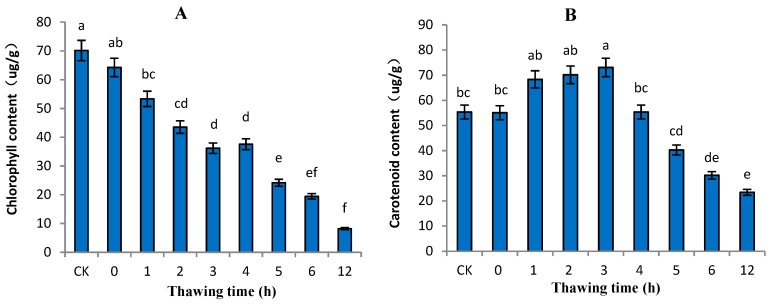
Changes in chlorophyll (**A**) and carotenoid contents (**B**) in “Ruaner” pear during the freezing–thawing stage. Different letters indicated significant differences between thawing time treated and control (Duncan’s multiple range test, *p* < 0.05).

**Figure 2:**
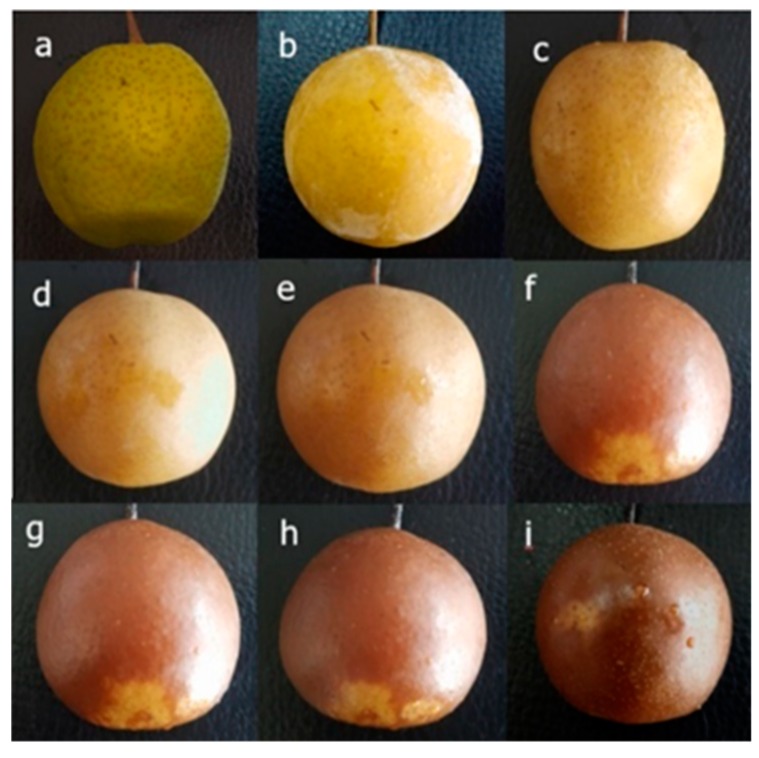
Photographs of “Ruaner” pear during the freezing–thawing stage (**a**) ck, (**b**) 0 hours after thawing (HAT), (**c**) 1 HAT, (**d**) 2 HAT, (**e**) 3 HAT, (**f**) 4 HAT, (**g**) 5 HAT, (**h**) 6 HAT, (**i**) 12 HAT.

The color of a fruit’s peel is an important factor that determines its marketability [21,22]. Chlorophyll, carotenoids, and anthocyanins are the main pigments in red fruit peels [23], while chlorophyll and carotenoids are the main pigments in pear peels [24]. Chlorophyll degradation is an important contributor to the changes in pigment composition that typically occur in fruit peel at the onset of ripening [25]. In this study, the changes in the chlorophyll and carotenoids in “Ruaner” pear peel were dramatic. During the freezing–thawing period of the “Ruaner” pear, the chlorophyll content decreased significantly (from 64.27 to 8.18 µg/g) within 0–12 HAT. The carotenoid content increased between 0 and 3 hours after thawing (HAT), at which point it peaked with a value of 73.08 µg/g (Figure 1); then, it decreased between 3 and 12 HAT, ending with a carotenoid composition that was lower than its initial value (0 HAT) and that of control pear fruit. The results show that during the earlier stage of ‘Ruaner’ pear freezing–thawing, the color of “Ruaner” pear peel changed from yellow-green to yellow (Figure 2), which is the result of chlorophyll degradation and carotenoid synthesis.

### 2.2. Total Phenolic and Total Flavonoid Contents of “Ruaner” Pear

**Figure 3:**
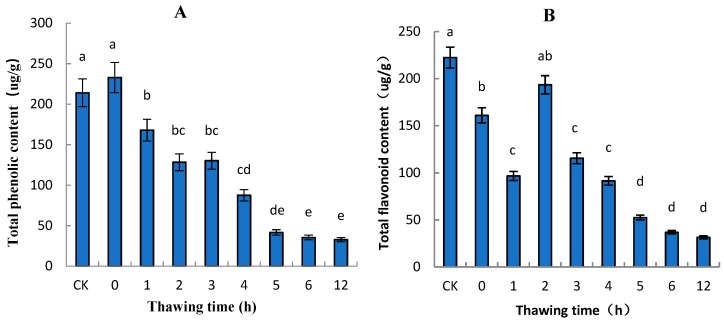
Changes in total phenolic (**A**) and flavonoid content (**B**) in “Ruaner” pear during the freezing–thawing stage. Different letters indicated significant differences between thawing time treated and control (Duncan’s multiple range test, *p* < 0.05).

The phenolics in fruits such as pear can determine the extent of lignification, flavor, and astringency, and the tendency to undergo oxidative browning. In this study, during the thawing stage of “Ruaner” pear, the color of pear pericarp gradually became brown (Figure 1), and the total phenolic content decreased. At 0 HAT, total phenolic content was 232.83 µg/g (Figure 3), and from 0 to 5 HAT, the total phenolic content dramatically decreased to 41.75 µg/g. From 5 to 12 HAT, the total phenolic content did not significantly change. The total flavonoid content of the control was 222.40 µg/g, while that of the 0 HAT and 1 HAT pear was 160.89 and 96.71 µg/g, respectively. From 2 to 6 HAT, the total flavonoid content dramatically decreased from 193.53 to 36.84 µg/g, and from 6 to 12 HAT, the total flavonoid content did not significantly change. Previous studies have shown that most of the antioxidants in fruits are gradually lost after harvest [24]. In this study, the total phenolic and total flavonoid content generally decreased.

### 2.3. Variation in Firmness, Water Content, Soluble Solids Content (SSC), and Titratable Acidity (TA) of “Ruaner” Pear

**Figure 4:**
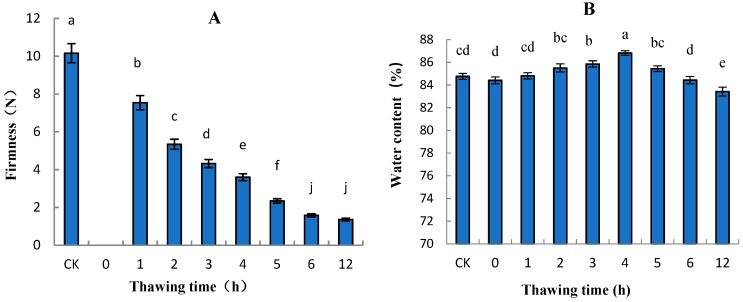
Changes in firmness (**A**) and water content (**B**) in “Ruaner” pear during the freezing–thawing stage. At 0 h, the fruit is frozen solid, so its firmness cannot be determined. Different letters indicated significant differences between thawing time treated and control (Duncan’s multiple range test, *p* < 0.05).

Firmness is a good indicator of a fruit’s maturity, so the degree of firmness can be used to gauge the remaining shelf life. Thus, assessing fruit maturity during postharvest storage is very important. In this study, significant differences in firmness were found among “Ruaner” pears at different freezing–thawing after-ripening stages. During the “Ruaner” pear freezing–thawing period, the firmness decreased significantly between 1 and 6 HAT (from 7.54 to 1.58 N), and from 6 to 12 HAT, the degree of firmness did not significantly change (Figure 4). The firmness of the control pear was 10.16 N (Figure 4). Water content is a very important indicator of pear quality. The water content tended to rise and then decrease, peaking at 4 HAT with a water content of 86.20%. The water content decreased to the lowest value at 12 HAT, at which time it was lower than the value at 0 HAT and that of the control pear fruit (Figure 4).

**Figure 5:**
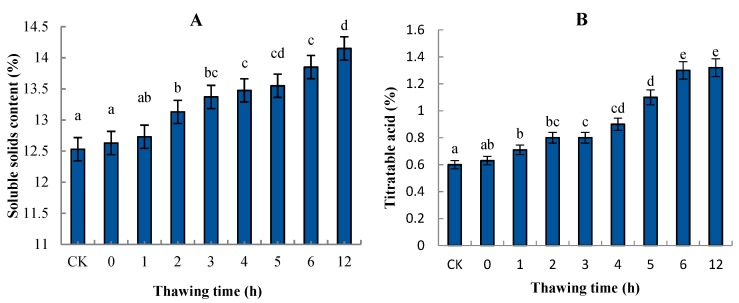
The change in soluble solids content (SSC) (**A**) and titratable acidity (TA) (**B**) content in “Ruaner” pear during the freezing–thawing stage. Different letters indicated significant differences between thawing time treated and control (Duncan’s multiple range test, *p* < 0.05).

SSC and TA are important indicators of maturity and quality in many fruit species. In this study, the SSC of the control pear was 12.53%. During “Ruaner” pear freezing–thawing, the SSC increased significantly from 1 to 12 HAT (from 12.63% to 14.15%). The TA increased significantly between 0 and 6 HAT (from 11.2% to 14.3%); from 6 to 12 HAT, TA did not significantly change (Figure 5).

### 2.4. Sugar and Organic Acid Contents of “Ruaner” Pear

**Figure 6:**
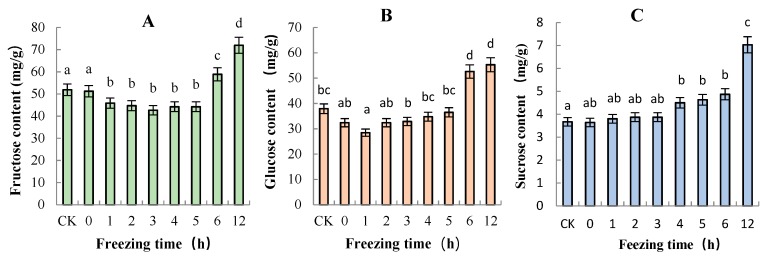
Changes in soluble sugar (**A**: fructose, **B**: glucose, **C**: sucrose) content in “Ruaner” pear during the freezing–thawing stage. Different letters indicated significant differences between thawing time treated and control (Duncan’s multiple range test, *p* < 0.05).

**Figure 7:**
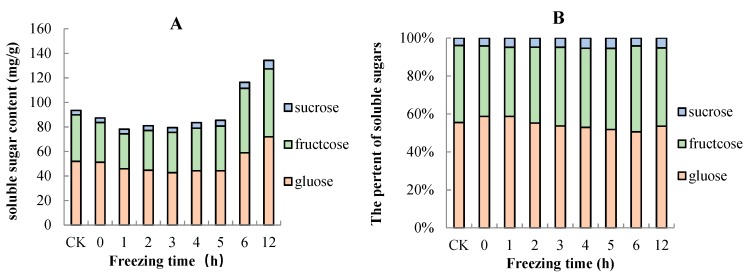
Changes in total soluble sugar content (**A**) and percentage composition of soluble sugars (**B**) in “Ruaner” pear during the freezing–thawing stage.

Three different sugars of “Ruaner” pear were measured by HPLC, namely, fructose, sucrose, and glucose. The concentrations of glucose, fructose, and sucrose generally increased during the freezing–thawing period (Figure 6). The major soluble sugars were fructose and glucose, which had ranges of 42.66–71.96 mg/g and 28.47–55.31 mg/g, accounting for 50.59–58.70% and 36.43–45.22% of the total soluble sugar, respectively (Figure 7). Of the three sugars analyzed, the sucrose concentration was the lowest and ranged from 3.64 to 7.03 mg/g, accounting for 3.92–5.42% of the total soluble sugar (Figure 7). The concentrations of total soluble sugar decreased between 0 and 1 HAT. After that, its content did not significantly change from 2 to 5 HAT. From 5 to 12 HAT, total soluble sugar content continuously increased from 85.36 to 134.30 mg/g. The concentrations of fructose and glucose decreased significantly from 0 to 1 HAT, and from 2 to 5 HAT, they did not significantly change. From 5 to 12 HAT, the two sugars increased rapidly, with the contents increased from 44.23 to 71.96 mg/g and from 36.50 to 55.31 mg/g, respectively. Moreover, the sucrose concentration in “Ruaner” pear did not tend to decrease, and it continuously increased until 12 HAT (from 3.64 to 7.03 mg/g). 

**Figure 8:**
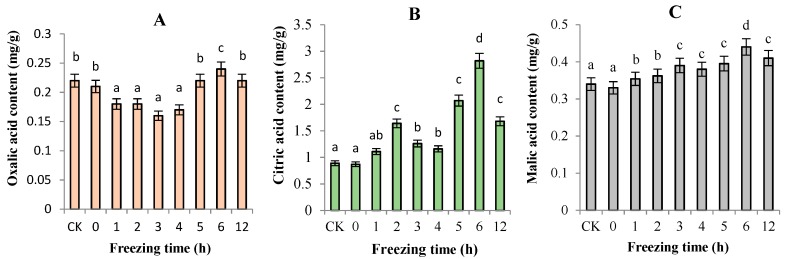
Changes in organic acid (**A**: oxalic acid, **B**: citric acid, **C**: malic acid) content in “Ruaner” pear during the freezing–thawing stage. Different letters indicated significant differences between thawing time treated and control (Duncan’s multiple range test, *p* < 0.05).

**Figure 9:**
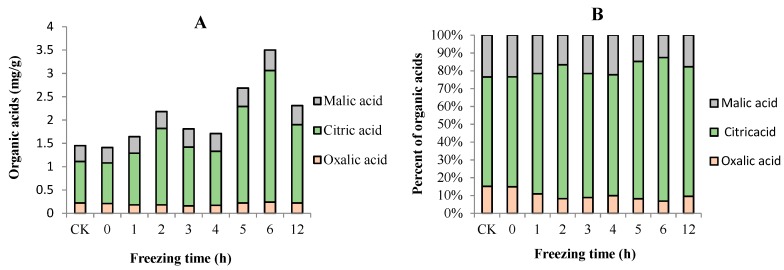
Changes in total organic acid content (**A**) and percentage composition of organic acids (**B**) in “Ruaner” pear during the freezing–thawing stage.

Although the organic acid content in fruit is regarded as one of its most commercially important quality traits when assessed by the consumer, relatively little is known about the change in organic acid in “Ruaner” pear. In this research, organic acid composition and concentration in “Ruaner” pear samples were analyzed by HPLC. There were great differences in the concentrations of individual organic acids, although their tendencies were the same during the ripening period. Figure 8 shows the concentration changes in these organic acids in “Ruaner” pears. Citric acid is the most abundant organic acid in the “Ruaner” pear; in the samples tested, citric acid ranged from 0.87 to 2.82 mg/g and accounted for 61.70–80.57% of the total acid (Figure 8 and Figure 9). The malic acid and oxalic acid contents were lower and ranged from 0.33 to 0.44 mg/g and from 0.16 to 0.24 mg/g, accounting for 15.57–23.45% and 8.19–15.17% of the total organic acid content, respectively (Figure 8 and Figure 9). The concentration of total organic acid increased from 1.41 to 2.18 mg/g between 0 and 2 HAT, and then it decreased from 2.18 to 1.71 mg/g between 2 and 4 HAT. Then, from 4 to 6 HAT, the citric acid concentration increased from 1.17 to 3.50 mg/g and then decreased at 12 HAT (Figure 8). The citric acid concentration increased from 0.87 to 1.64 mg/g between 0 and 2 HAT, after which it decreased from 1.64 to 1.16 mg/g between 2 and 4 HAT. From 4 to 6 HAT, the citric acid concentration increased from 1.16 to 2.82 mg/g, and then it decreased (Figure 8). The concentration of oxalic acid decreased from 0.21 to 0.16 mg/g between 0 and 3 HAT, but from 3 to 6 HAT, it increased rapidly from 0.16 to 0.24 mg/g (Figure 8). The malic acid variation trend was quite similar to that of citric acid. The malic contents continuously increased between 0 and 6 HAT, after which it then decreased until 12 HAT (Figure 8).

## 3. Discussion

Most fruits are stored at about 0 °C to maintain freshness, but this method of preservation is limited by storage costs because it needs controlled temperature and (or) gases composition of storage. The natural freezing method is a good traditional storage method in the North of China, where frozen pear is a delicious specialty and the average temperature is below −20 °C in winter. Under these climatic circumstances, fresh pears are stored outdoors for more than 10 days, leading to the formation of frozen pears. If their temperature is warmed to room temperature before eating, frozen pears become soft and very delicious. However, the physical–chemical composition and quality-related changes that occur during the thawing process are yet to be determined.

### 3.1. Variation in Total Polyphenols and Total Flavonoids in “Ruaner” Pear

Flavonoids and polyphenols play very important roles in plants and human health because they are co-pigments that determine fruit color and have high antioxidant properties [25,26]. Previous studies have shown that most of the antioxidants in fruits are gradually lost after harvest [22]. In our research, as the thawing time increase, phenolic content generally decreased. Between 5 and 12 HAT, their contents were lower and stable. Compared with fresh “Ruaner” pear, the content of polyphenols in frozen pear (5 HAT) was decreased by about 81.31%, and the flavonoid content in “Ruaner” pear decreased by about 77.27%. This suggests that the antioxidant properties of flavonoids and polyphenols in “Ruaner” pear decreased between 5 and 12 HAT.

Recent studies have shown that after chilling, injured “Nanguo” pears (Ussurian pear variety) undergo browning [27,28], and the reason that the pear peel is brown is that polyphenols are oxidized to the corresponding quinones by polyphenol oxidases (PPO) [26]. In our study, from 3 to 12 HAT, the color of “Ruaner” pears changed from yellow to brown. The enhanced brown color on the fruit surface may be attributed to polyphenol degradation and an increase in quinones (Figure 1). However, in normally developing cells, PPO and phenolic compounds are separated from each other in different cell locations by cellular membranes [29]. We infer that during the freezing–thawing stage, the cell membrane of “Ruaner” pear must be damaged starting at 3 HAT and that, as a result, the cell membrane permeability increases, causing polyphenol oxidases and polyphenols to mix and react with each other.

### 3.2. Variation in Firmness, Soluble Solid Content (SSC) and Titratable Acidity (TA) of “Ruaner” Pear

Before eating frozen “Ruaner” pear, it is warmed to room temperature. After thawing, the taste of the frozen “Ruaner” pear becomes soft, juicy, and mildly sour and sweet, depending on the changes in quality parameters, such as firmness, water content, SSC, and TA. In this research, the change in firmness was remarkable (Figure 2) during the freezing–thawing stage. The firmness decreased significantly between 1 and 6 HAT, while from 6 to 12 HAT, the firmness did not significantly decrease. The main reason for the decreased firmness is the melting of the ice in the fruit. According to previous studies, the conversion of starch and pectin substances is the reason for fruit becoming soft [7,10,30]. This means that these processes were completed before 6 HAT the SSC increased significantly from 1 to 12 HAT. The fact that the 12-HAT samples had the highest SSC value might be associated with the transformation of pectin substances and starch hydrolysis [30]. During the “Ruaner” pear thawing process, water content increased between 0 and 4 HAT. The main cause may be that the fruits adsorb air moisture while thawing. Later, the water content decreased, which may be the result of water loss from the fruits due to skin damage during the thawing process. Lastly, for the TA measurement, 6-HAT samples had a mean value that was 1.2-fold higher than the control group. This result is consistent with previous research results [3]. Further research will be conducted to explore the reasons for the TA increase. The data show that freezing–thawing significantly changed firmness, water content, SSC, and TA in “Ruaner” pears, especially at 4 HAT, at which time “Ruaner” pears had moderate hardness, high water content, high soluble solids, and low acid content. Therefore, 4 HAT is the best time for pears in terms of both table and processing quality.

### 3.3. Soluble Sugars and Organic Acid Contents in “Ruaner” Pear

Fruit postharvest ripening is a complex physiological and biochemical process that involves changes in peel color, fruit texture, sugar acid components, aroma, and so on. The fruit’s taste is largely affected by the composition and concentration of soluble sugars and organic acids. In our study, soluble sugars and organic acids composition and concentration in “Ruaner” pear were analyzed by subjecting flesh samples to HPLC during the thawing process. The major soluble sugars were fructose and glucose, which made up 50.59–58.70% and 36.43–45.22% of the total soluble sugars, respectively. These were followed by sucrose, which accounted for 3.92–5.42% of the total soluble sugar. Previous studies have identified fructose, glucose, sucrose, and sorbitol deposits in the flesh of pear fruit [31]. Sorbitol was not detected in the test cultivars, possibly because the sorbitol content was lower than the threshold of instrumental detection. Alternatively, it might have degraded. The concentrations of fructose, glucose, sucrose, and total soluble sugar changed little from 0 to 5 HAT. However, from 5 to 12 HAT, the concentrations continuously and dramatically increased; in other words, 5–12 HAT was the period with the highest sugar content during the freezing–thawing period. Vacuoles contribute to the storage of sugars [32]. During the freezing–thawing stage, damage to the vacuolar membrane leads to extravasation of soluble sugar with cell fluid, and this may be a factor that increases soluble sugar. However, from 5 to 12 HAT, fructose, glucose, sucrose, and total soluble sugar continuously and dramatically increased, and we speculate that the degradation of starch may be the main factor for the increase in soluble sugar. Further verification is still needed to investigate the mechanism of soluble sugar’s increase. In addition, in our research, the SSC remained unchanged, while the total soluble sugar dramatically increased from 5 to 12 HAT, which may be due to the loss of other soluble substances, such as vitamins and minerals and so on.

Organic acids are also important substances that determine the taste of the fruit. Previous studies have shown that the major components of organic acids in the pear fruit are malic and citric acid [16,33]. In our study, there were three organic acids in the flesh of the “Ruaner” pear: oxalic acid, citric acid, and malic acid in the flesh of the “Ruaner” Pear. Citric acid was the most abundant organic acid in “Ruaner” pear, accounting for 61.70–80.57% of the total organic acid, followed by malic and oxalic acid, accounting for 15.17–23.45% and 6.86–15.00% of the total organic acids, respectively. The concentrations of the three individual organic acids greatly differed, but the tendency was the same from 4 to 12 HAT, during which the acid content tended to rise and then decrease, peaking at 6 HAT. In other words, 6 HAT was the period with the highest acid content during the freezing–thawing period.

## 4. Materials and Methods

### 4.1. Plant Material

“Ruaner” pear fruits were picked from a commercial orchard in Anning District, Lanzhou, Gansu Province, Northwest China (36°06′ N, 103°42′ E) on 1 October in 2017 and 2018. Pear fruits of similar size and color were selected for sampling. After harvest, the fruits were transported to the laboratory for selection and experimental treatment. Uniform fruit without visible signs of defects were selected as the experimental materials. The fruits were left at room temperature for 3 days then divided into two groups: the control group and the treatment group. Fruits in the treatment group were carefully put into a plastic box, which was then sealed tightly and frozen at −20 °C for 7 days. After treatment, all of the fruits were transferred to room temperature for thawing. During the thawing stage, the fruits were sampled at 0, 1, 2, 3, 4, 5, 6, and 12 h for further analysis. 4 pears were peeled with a hand peeler (about 1 mm thickness). Approximately 1.00 g of peel and 5.00 g flesh were taken from each pear. The peels and the flesh of the 4 pears were pooled. Pigments, polyphenols, and flavonoids were extracted from the fruit peel, and water content, SSC, TA, sugars, and organic acids were analyzed from fruit fresh without the peel. Each component was replicated 3 times (*n* = 3).

### 4.2. Pigment Extraction And Quantification

The peel from “Ruaner” pear (0.5 g) was used in the extraction and ground to a fine homogenate. Chlorophyll and carotenoids were extracted for 72 h using acetone (80%) in the dark. The absorbances at 647 and 664 nm were determined using a spectrophotometer (UC-2450, Shimadzu, Japan) and were used to calculate the concentration of chlorophyll and carotenoid, as described by Chen and Wang (2002) [34]. chlorophyll and carotenoid contents were calculated using the equation: Ct = 20.2 A645 + 8.02 A663 and Ck = 4.7 A440 − 0.27Ct.

### 4.3. Total Polyphenol and Flavonoid Extraction and Quantification

The total phenolic were extracted as described by Wolfe et al. (2003) [35]. “Ruaner” pear peel (10 g) was homogenized for 3 min with 100 mL of chilled 80% acetone solution. The homogenate was filtered through Whatman No. 1 filter paper in a Buchner funnel under vacuum. The solids were scraped into 150 mL of 80% acetone and homogenized for 3 min before refiltration. The filtrate was recovered and evaporated using a rotary evaporator (Büchi, Flawil, Switzerland) at 45 °C until less than 10% of the initial volume remained. The extract was brought to a volume of 50 mL with distilled water then frozen at −40 °C until analysis. All of the compounds were extracted three times. The total phenol content in the peel was determined using the Folin–Ciocalteu colorimetric method [36]. Spectrophotometer (UC-2450, Shimadzu, Japan) was used for the absorbance measurements to determine the total flavonoid content, and total phenolic content was expressed as microgram gallic acid equivalents (GAE) per gram of fresh material. The total flavonoid was extracted as described by Wolfe et al. (2003) [35]. The extract (0.25 mL) was added to a tube containing 1.25 mL of distilled water. To the mixture was added 0.075 mL of 5% sodium nitrite solution. Then, 10% aluminum chloride (0.15 mL) was added. After 6 min, 1 M sodium hydroxide (0.5 mL) was added, and the mixture was diluted with distilled water (0.275 mL). Spectrophotometer (UC-2450, Shimadzu, Japan) was used for the absorbance measurements to determine the total phenolic contents. The absorbance was measured at 510 nm, and the total flavonoid content was expressed as micrograms quercetin equivalents per gram of fresh material.

### 4.4. Firmness, Water Content, Soluble Solid Content (SSC) and Titratable Acidity (TA) Determination

A GY-4 (China) was applied to measure fruit firmness. A 0.5 cm^2^ probe was chosen for our measurements. The penetration depth and penetration rate were 5 mm and 10 mm/s. Measurements were made at the equator of the fruit after removal of a 1 mm thick slice of peel, and the maximum force (N) was used as the measurement of fruit firmness.

The pear flesh (5.00 g) was placed in a drying box and transported to the laboratory as quickly as possible in order to minimize water loss due to evaporation. Samples were weighed at two stages: immediately after sampling (fresh weight) and after drying in an incubator and then oven-drying at 70 °C for 24 h (repeated 4 times). The water content was then calculated from the following formula: Water content = (Fresh weight − Dry weight)/5 × 100.

The concentration of SSC was measured in the juice extracted from three different points at the equator of one fruit by PAL-1 (ATAGO China), and the mean values were used to indicate the SSC of the pear.

The TA analysis was carried out using the LMBG method (1983) [37]. Titratable acidity (TA) of the supernatant was determined by titration with 0.01 mol L^−1^ NaOH to pH 8.1. There were 4 replications with 4 fruits in each treatment.

### 4.5. Extraction, Purification, and Isolation of the Sugars and Organic Acids

The sugars and organic acids were extracted as described by Liu et al. (2013) [12]. The fruit flesh (5.00 g) from four “Ruaner” pears was used in the extraction and ground to a fine homogenate. An aliquot (25 mL) of the resultant supernatant was used for HPLC analysis. The sugar (fructose, glucose, and sucrose) and organic acid (malic, citric, and oxalic) contents were analyzed by HPLC (Shimadzu, Kyoto, Japan). The separation of soluble sugars was carried out using a ZORBAX NH_2_ with a 5 μm column from Du Pont Co. (4.6 × 250 mm) operated at 40 °C. The mobile phase was acetonitrile (Chromatographic grade) and bi-distilled water (7/3), and the flow rate was 1.0 mL/min. The total run time was 25 min, and an RI detector (Shimadzu, Kyoto, Japan) was used to monitor the soluble sugars, as described by Liu et al. [12]. Organic acids were analyzed with HPLC using an IC PAK TM ION exclusion column (300 mm × 7.8 mm) (Waters, Milford, DE, USA) associated with a PDA HPLC (Shimadzu, Kyoto, Japan) detector set at 210 nm, as described by Liu et al. [12]. The column temperature was set at 40 °C. The elution solvent was 0.01 mM sulfuric acid (Chromatographic grade) in bi-distilled water at a flow rate of 0.5 mL/min. The duration of the analysis was 30 min. Standards were fructose, glucose, sucrose and malic, citric, oxalic (Sigma Chemical, St. Louis, MO, USA). Sugars and organic acids were expressed as mg/g FW.

### 4.6. Statistical Analysis

Statistical analysis was conducted with SPSS 17.0. One-way ANOVA was used for the analysis of pigment, sugar, organic acid, and color parameter concentration. Differences between the sampling dates were estimated with the Duncan test (*p* < 0.05).

## 5. Conclusions

In this study, physical–chemical composition and quality-related changes in “Ruaner” pear were determined during freezing–thawing stage. The results indicate that the color of “Ruaner” pear peel changed from yellow–green to yellow and then brown. The chlorophyll content and TA decreased significantly throughout 0–12 h period. The carotenoid content tended to rise and then decrease, peaking at 3 HAT. The and SSC, firmness and total phenolic and flavonoid content all generally decreased. The major soluble sugars were fructose and glucose, which ranged of 42.66–71.96 mg/g and 28.47–55.31 mg/g, accounting for 50.59–58.70% and 36.43–45.22% of the total soluble sugar respectively. Citric acid was the most abundant organic acid in “Ruaner” pear during freezing–thawing, accounting for 0.87 to 2.82 mg/g and accounted for 61.70–80.57% of the total acid. The data suggest that freezing–thawing significantly changes firmness, water content, SSC, and TA in “Ruaner” pears. At 3–4 HAT, “Ruaner” pear have moderate hardness, low acid content, and higher water, total phenolic, total flavonoid, and soluble solid contents. Thus, 3–4 HAT is the best time for these pears in terms of both table and processing quality.
